# Long COVID-19 and Postural Orthostatic Tachycardia Syndrome- Is Dysautonomia to Be Blamed?

**DOI:** 10.3389/fcvm.2022.860198

**Published:** 2022-03-09

**Authors:** Karan R. Chadda, Ellen E. Blakey, Christopher L. -H. Huang, Kamalan Jeevaratnam

**Affiliations:** ^1^Cambridge University Hospitals NHS Foundation Trust, Cambridge, United Kingdom; ^2^Physiological Laboratory, University of Cambridge, Cambridge, United Kingdom; ^3^Department of Biochemistry, University of Cambridge, Cambridge, United Kingdom; ^4^Faculty of Health and Medical Sciences, University of Surrey, Guildford, United Kingdom

**Keywords:** long COVID, tachycardia, postural orthostatic tachycardia syndrome, dysautonomia, arrhythmia

## Abstract

While the increased arrhythmic tendency during acute COVID-19 infection is recognised, the long-term cardiac electrophysiological complications are less well known. There are a high number of patients reporting ongoing symptoms post-infection, termed long COVID. A recent hypothesis is that long COVID symptoms could be attributed to dysautonomia, defined as malfunction of the autonomic nervous system (ANS). The most prevalent cardiovascular dysautonomia amongst young people is postural orthostatic tachycardia syndrome (POTS). Numerous reports have described the development of POTS as part of long COVID. Possible underlying mechanisms, although not mutually exclusive or exhaustive, include hypovolaemia, neurotropism, inflammation and autoimmunity. Treatment options for POTS and other long COVID symptoms are currently limited. Future research studies should aim to elucidate the underlying mechanisms of dysautonomia to enable the development of targeted therapies. Furthermore, it is important to educate healthcare professionals to recognise complications and conditions arising from COVID-19, such as POTS, to allow prompt diagnosis and access to early treatment.

## Introduction

The Hubei Integrated Chinese and Western Medicine hospital in Wuhan reported a clustered point-source outbreak of pneumonia, of unknown viral origin toward the end of December 2019. Within 30 days, the rapid geographic spread of the disease, subsequently called Coronavirus Disease 2019 (COVID-19), implied propagation by human-to-human transmission (1, 2). A pandemic was declared on March 11th 2020, by World Health Organization (WHO) (3). As of November 2021, the disease has caused over 5 million deaths worldwide (4). The disease primarily associated with respiratory pathology, also has well-known cardiac complications, including acute coronary syndrome, myocarditis, heart failure and arrhythmia (5). Indeed, in one cohort of hospitalised COVID-19 patients, heart palpitations were part of the presenting complaint in 7.3% of patients (6). An early study on patients hospitalised with COVID-19 in Wuhan showed the incidence for cardiac arrhythmic events was 17%, rising to 44% in those admitted to the intensive care unit (7). A more recent meta-analysis demonstrated an arrhythmic incidence of 19% in patients with COVID-19, and these patients had an increased risk of poor outcomes (8). While the increased arrhythmic tendency during acute infection is recognised, the long-term cardiac complications are less clear and remain to be reported. The following review is focussed on the associations between long COVID and tachycardia, specifically postural orthostatic tachycardia syndrome (POTS), the potential aetiologies and a discussion on the future direction of research.

## Long COVID

Although the symptoms and complications of acute COVID-19 infection are becoming better understood, more research is required to understand the longer-term impact on patients. Mortality rates and hospital admissions have declined due to vaccination rollouts and evolving treatments (9, 10), but there are a high number of patients reporting ongoing symptoms post-infection, termed long COVID (11, 12). For example, one study showed a persistence of symptoms in 87.4% of 143 patients with COVID-19, with fatigue being the most common (53.1%) (13). A prospective cohort study with a larger sample size of 1,733 and longer follow-up showed 76% of patients reported at least one persistent symptom after COVID-19 infection (14). Thus, growing clinical and scientific evidence suggests long-lasting effects of COVID-19 impact multiple organ systems. Symptoms can include palpitations, chronic cough, shortness of breath, chest pain, cognitive dysfunction, fatigue, arthralgia, and overall decline in quality of life (11, 15, 16). Arguably, this is not surprising, as similar patterns have been observed in previous coronavirus infections, such as the severe acute respiratory syndrome (SARS) and Middle East respiratory syndrome (MERS) outbreaks (15). There is clearly a clinical need to understand the pathophysiology behind long COVID, in order to develop targeted therapeutic interventions.

## “Post-COVID-19 Tachycardia Syndrome”

From a cardiovascular point of view, recent papers have highlighted an association between orthostatic intolerance (OI), including orthostatic hypotension (OH) and postural orthostatic tachycardia syndrome (POTS), with long COVID (17–19). OH is defined by a systolic or diastolic blood pressure decrease of 20 mmHg or 10 mHg, respectively, within 3 min of standing and it is the most common autonomic manifestation of long COVID (18, 20, 21). Studies suggest the prevalence of OH with long COVID may range between 10% (17) up to 41% (20, 21). Furthermore, tachycardia has been found to be a common symptom associated with long COVID, with 25–50% of patients in a tertiary post-COVID multidisciplinary team clinic reporting persistent tachycardia or palpitations (22). It has been suggested that persistent tachycardia seen in long COVID, labelled “post-COVID-19 tachycardia syndrome,” may present as inappropriate sinus tachycardia or POTS (22). Inappropriate sinus tachycardia occurs when there is a higher heart rate response or faster resting rate than necessary for the current physiological demand (23). At present, there is limited data available on this in the context of long COVID. POTS is defined as a persistent increase in heart rate of at least 30 beats per minute within 10 min of standing and this can present with symptoms of palpitations, chest pain and exercise or orthostatic intolerance (24). Numerous reports have described the development of POTS as part of long COVID (19, 25–27) although the aetiology remains debated.

## Mechanisms of Dysautonomia Leading to Postural Orthostatic Tachycardia Syndrome

**FIGURE 1:**
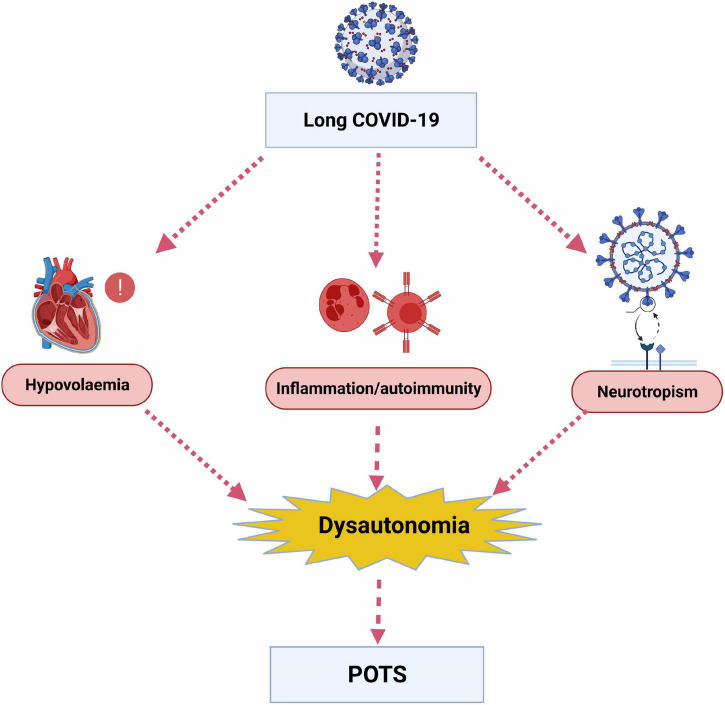
Potential underlying mechanisms by which long COVID leads to dysautonomia and postural orthostatic tachycardia syndrome (POTS). (Created with BioRender.com).

A recent hypothesis is that long COVID symptoms could be attributed to dysautonomia (16, 28). Dysautonomia can be defined as malfunction of the autonomic nervous system (ANS) (29), and it can be acute or chronic and progressive or reversible (28). Many symptoms can manifest from dysautonomia, including fatigue, heart rate variability (HRV) dysfunction and orthostatic hypotension (28). The most prevalent cardiovascular dysautonomia amongst young people is postural orthostatic tachycardia syndrome (POTS) (30). The interaction of the autonomic nervous system and cardiac rhythm abnormalities has been previously described (31) but there remains limited data on long COVID, dysautonomia and the aetiology of POTS. Possible underlying mechanisms, although not mutually exclusive or exhaustive, include hypovolaemia, neurotropism, inflammation and autoimmunity (Figure 1) (24, 28).

### Hypovolaemia

Firstly, it has been suggested that POTS occurs due to decreased blood volume and deconditioning of the heart, which subsequently increases cardiac sympathetic nervous system (SNS) output (24, 32, 33). COVID-19 can be associated with fever and nocturnal sweating, leading to hypovolaemia, and prolonged bed rest, leading to deconditioning and cardiac atrophy. Furthermore, it has been shown that patients with POTS had a lower plasma volume compared to controls (34). The rein-angiotensin-aldosterone system (RAAS) may have a role in the aetiology of POTS as despite lower plasma volumes, it has been shown that patients with POTS had lower levels of plasma renin and aldosterone (34–36). This RAAS deficit could be due to partial renal sympathetic denervation (34). SARS-CoV-2 has been implicated in binding and down-regulating the ACE2 receptor (37), and introducing a RAAS imbalance (38). It has also been proposed that SARS-CoV-2 infection causes a deficit of cellular furin, leading to dysfunction of the epithelial sodium channel (ENaC) and changes in fluid balance that activates the RAAS (39).

Thus, the persistent tachycardia is a physiological compensation against the smaller stroke volume of the deconditioned heart (40) and those that are more predisposed to the condition have smaller hearts (32). Interestingly, POTS is observed in patients with chronic fatigue syndrome (41) and a prevent study demonstrated many patients with chronic fatigue syndrome had small hearts (42).

### Neurotropism

Secondly, a suggested and more direct cause of POTS could be related to the spread of COVID-19 to the central nervous system (CNS) by neural propagation, causing direct damage on nerves or indirect damage via a cytokine storm (28). Cerebrospinal spinal fluid (CSF) in patients with acute COVID-19 have shown the presence of the virus (37) and PET scans have shown hypometabolism in the olfactory bulb as well as extension to other brain areas at the post-viral stage of COVID-19 disease (43). Similar to other coronaviruses, the COVID-19 virus binds to the ACE2 receptor on host membranes by using a spike protein S1 (37). ACE2 receptors are highly expressed in the olfactory epithelium and thus a proposed propagation route is via the nose through the cribriform plate of the ethmoid bone to the olfactory epithelium (43), which could be responsible for the hyposmia observed with COVID-19 infection. Thus, it has been proposed that SARS-Cov2 could infect and damage extracardiac postganglionic SNS, leading to dysautonomia in a similar way to neuropathic POTS. This disruption could lead to tachycardia due to splanchnic venous pooling or reduced mesenteric vasoconstriction during orthostasis (24).

### Inflammation and Autoimmunity

Thirdly, heart rate variability (HRV) can be used as a marker of dysautonomia (44) and HRV has been shown to have an inverse relationship to inflammation in a recent meta-analysis (45). In addition, the role for the autoimmune system and inflammation in the aetiology of POTS is supported by the increased antibodies and incidence of autoimmune conditions seen in patients with POTS compared to controls (46). COVID-19 infection can cause a cytokine storm and inflammation that could trigger chronic neuronal dysregulation (47). One mechanism could be via the development of autoantibodies which cross-react with autonomic ganglia as part of acute and long-term COVID-19 infection, and lead to dysautonomia and POTS. Li et al. showed patients with POTS had high levels of autoantibodies which cross-reacted with muscarinic and beta receptors (48). The role of the immune system in dysautonomia and long COVID-19 could have implications for treatment options as 75% of patients with POTS were positive for α3-AChR antibodies that could respond to immunotherapy (49).

## Management of Postural Orthostatic Tachycardia Syndrome

Currently, conservative treatment options include graduated exercise programmes, avoiding triggers, high fluid and salt intake, compression stockings and cognitive behavioural therapy (50, 51). Pharmacological options are generally unlicensed for this indication but aim to correct several physiological parameters. These include agents, such as fludrocortisone, erythropoietin and desmopressin, to increase blood volume, β-blockers or ivabradine to reduce tachycardia, midodrine, methylphenidate or octreotide to induce vasoconstriction and pyridostigmine to facilitate synaptic transmission (34, 50, 52). Evidently, further research is needed to elucidate the underlying mechanisms of dysautonomia to direct targeted treatment options. Indeed, in the absence of novel therapies, it is a viable option to repurpose drugs (53) once a better mechanistic understanding of long COVID and dysautonomia is established.

Central to the management of POTS is prompt recognition as the correct diagnosis of POTS can be delayed by several years (54). Unfortunately, POTS is often misdiagnosed as patients present with many non-specific symptoms, and the significance of the symptoms, such as asymptomatic tachycardia in an otherwise well patient, may not be appreciated. From a physiological perspective, persistent tachycardia will inevitably lead to remodelling of the heart and predispose to cardiac failure in the future. Thus, persistent tachycardia as a sole symptom still warrants consideration of further investigation and treatment. The impact of long COVID-19 on the incidence cardiac failure secondary to persistent tachycardia remains to be seen but should be considered in future research studies.

## Conclusion

Unfortunately, given the number of people infected with COVID-19, there is likely to be an emergence of various long COVID-19 syndromes presenting to both primary and secondary care, such as patients with POTS. Thus, there is clearly a need for future studies to investigate the cause of long COVID-19 symptoms. One avenue would be to better understand the aetiology of dysautonomia to enable the development of therapies that specifically target the underlying pathology. Meanwhile, it is important to educate healthcare professionals to recognise complications and conditions arising from COVID-19, such as POTS, to allow prompt diagnosis and referral to enable early treatment access.

## Author Contributions

KC and EB wrote the first draft and review all subsequent draft. CH reviewed all drafts and supervised. KJ developed the idea, reviewed all drafts, and supervised. All authors contributed to the article and approved the submitted version.

## Conflict of Interest

The authors declare that the research was conducted in the absence of any commercial or financial relationships that could be construed as a potential conflict of interest.

## Publisher’s Note

All claims expressed in this article are solely those of the authors and do not necessarily represent those of their affiliated organizations, or those of the publisher, the editors and the reviewers. Any product that may be evaluated in this article, or claim that may be made by its manufacturer, is not guaranteed or endorsed by the publisher.

## References

[1] Coronaviridae Study Group of the International Committee on Taxonomy of Viruses. The species severe acute respiratory syndrome-related coronavirus: classifying 2019-nCoV and naming it SARS-CoV-2. *Nat Microbiol.* (2020) 5:536–44. 10.1038/s41564-020-0695-z 32123347 PMC7095448

[2] Takla M Jeevaratnam K. Chloroquine, hydroxychloroquine, and COVID-19: systematic review and narrative synthesis of efficacy and safety. *Saudi Pharm J.* (2020) 28:1760–76. 10.1016/j.jsps.2020.11.003 33204210 PMC7662033

[3] Cucinotta D Vanelli M. WHO declares COVID-19 a pandemic. *Acta Biomed.* (2020) 91:157–60. 10.23750/abm.v91i1.9397 32191675 PMC7569573

[4] Dong E Du H Gardner L. An interactive web-based dashboard to track COVID-19 in real time. *Lancet Infect Dis.* (2020) 20:533–4. 10.1016/S1473-309930120-1 32087114 PMC7159018

[5] Babapoor-Farrokhran S Gill D Walker J Rasekhi RT Bozorgnia B Amanullah A. Myocardial injury and COVID-19: possible mechanisms. *Life Sci.* (2020) 253:117723. 10.1016/j.lfs.2020.117723 32360126 PMC7194533

[6] Liu K Fang YY Deng Y Liu W Wang MF Ma JP Clinical characteristics of novel coronavirus cases in tertiary hospitals in Hubei Province. *Chin Med J (Engl).* (2020) 133:1025–31. 10.1097/CM9.0000000000000744 32044814 PMC7147277

[7] Wang D Hu B Hu C Zhu F Liu X Zhang J Clinical characteristics of 138 hospitalized patients with 2019 novel coronavirus-infected pneumonia in Wuhan, China. *JAMA.* (2020) 323:1061–9. 10.1001/jama.2020.1585 32031570 PMC7042881

[8] Pranata R Huang I Raharjo SB. Incidence and impact of cardiac arrhythmias in coronavirus disease 2019 (COVID-19): a systematic review and meta-analysis. *Indian Pacing Electrophysiol J.* (2020) 20:193–8. 10.1016/j.ipej.2020.08.001 32814094 PMC7428753

[9] Pawlowski C Lenehan P Puranik A Agarwal V Venkatakrishnan AJ Niesen MJM FDA-authorized mRNA COVID-19 vaccines are effective per real-world evidence synthesized across a multi-state health system. *Med (N Y).* (2021) 2:979.e–92.e. 10.1016/j.medj.2021.06.007 34223401 PMC8238652

[10] Dennis JM McGovern AP Vollmer SJ Mateen BA. Improving survival of critical care patients with coronavirus disease 2019 in England: a national cohort study, March to June 2020. *Crit Care Med.* (2021) 49:209–14. 10.1097/CCM.0000000000004747 33105150 PMC7803441

[11] Venkatesan P. NICE guideline on long COVID. *Lancet Respir Med.* (2021) 9:129. 10.1016/S2213-260000031-X PMC7832375 33453162

[12] Cooper SL Boyle E Jefferson SR Heslop CRA Mohan P Mohanraj GGJ Role of the renin-angiotensin-aldosterone and Kinin-Kallikrein systems in the cardiovascular complications of COVID-19 and long COVID. *Int J Mol Sci.* (2021) 22:8255. 10.3390/ijms22158255 34361021 PMC8347967

[13] Carfi A Bernabei R Landi F Gemelli Against COVID-19 Post-Acute Care Study Group. Persistent symptoms in patients after acute COVID-19. *JAMA.* (2020) 324:603–5. 10.1001/jama.2020.12603 32644129 PMC7349096

[14] Huang C Huang L Wang Y Li X Ren L Gu X 6-month consequences of COVID-19 in patients discharged from hospital: a cohort study. *Lancet.* (2021) 397:220–32. 10.1016/S0140-673632656-8 33428867 PMC7833295

[15] Nalbandian A Sehgal K Gupta A Madhavan MV McGroder C Stevens JS Post-acute COVID-19 syndrome. *Nat Med.* (2021) 27:601–15. 10.1038/s41591-021-01283-z 33753937 PMC8893149

[16] Goertz YMJ Van Herck M Delbressine JM Vaes AW Meys R Machado FVC Persistent symptoms 3 months after a SARS-CoV-2 infection: the post-COVID-19 syndrome? *ERJ Open Res.* (2020) 6:00542–2020. 10.1183/23120541.00542-2020 33257910 PMC7491255

[17] Buoite Stella A Furlanis G Frezza NA Valentinotti R Ajcevic M Manganotti P. Autonomic dysfunction in post-COVID patients with and witfhout neurological symptoms: a prospective multidomain observational study. *J Neurol.* (2022) 269:587–96. 10.1007/s00415-021-10735-y 34386903 PMC8359764

[18] Suresh K Alam MDU Satkovich E. COVID-19-associated dysautonomia. *Cureus.* (2021) 13:e17156. 10.7759/cureus.17156 34532186 PMC8435208

[19] Dani M Dirksen A Taraborrelli P Torocastro M Panagopoulos D Sutton R Autonomic dysfunction in ‘long COVID’: rationale, physiology and management strategies. *Clin Med (Lond).* (2021) 21:e63–7. 10.7861/clinmed.2020-0896 33243837 PMC7850225

[20] Shah B Kunal S Bansal A Jain J Poundrik S Shetty MK Heart rate variability as a marker of cardiovascular dysautonomia in post-COVID-19 syndrome using artificial intelligence. *Indian Pacing Electrophysiol J.* (2022). 10.1016/j.ipej.2022.01.004 35101582 PMC8800539

[21] Shouman K Vanichkachorn G Cheshire WP Suarez MD Shelly S Lamotte GJ Autonomic dysfunction following COVID-19 infection: an early experience. *Clin Auton Res.* (2021) 31:385–94. 10.1007/s10286-021-00803-8 33860871 PMC8050227

[22] Stahlberg M Reistam U Fedorowski A Villacorta H Horiuchi Y Bax J Post-COVID-19 tachycardia syndrome: a distinct phenotype of post-acute COVID-19 syndrome. *Am J Med.* (2021) 134:1451–6. 10.1016/j.amjmed.2021.07.004 34390682 PMC8356730

[23] Baruscotti M Bucchi A Milanesi R Paina M Barbuti A Gnecchi-Ruscone T A gain-of-function mutation in the cardiac pacemaker HCN4 channel increasing cAMP sensitivity is associated with familial inappropriate Sinus Tachycardia. *Eur Heart J.* (2017) 38:280–8. 10.1093/eurheartj/ehv582 28182231

[24] Goldstein DS. The possible association between COVID-19 and postural tachycardia syndrome. *Heart Rhythm.* (2021) 18:508–9. 10.1016/j.hrthm.2020.12.007 33316414 PMC7729277

[25] Kanjwal K Jamal S Kichloo A Grubb BP. New-onset postural orthostatic tachycardia syndrome following coronavirus disease 2019 infection. *J Innov Card Rhythm Manag.* (2020) 11:4302–4. 10.19102/icrm.2020.111102 33262898 PMC7685310

[26] Miglis MG Prieto T Shaik R Muppidi S Sinn DI Jaradeh S. A case report of postural tachycardia syndrome after COVID-19. *Clin Auton Res.* (2020) 30:449–51. 10.1007/s10286-020-00727-9 32880754 PMC7471493

[27] Umapathi T Poh MQW Fan BE Li KFC George J Tan JYL. Acute hyperhidrosis and postural tachycardia in a COVID-19 patient. *Clin Auton Res.* (2020) 30:571–3. 10.1007/s10286-020-00733-x 32970212 PMC7511524

[28] Barizien N Le Guen M Russel S Touche P Huang F Vallee A. Clinical characterization of dysautonomia in long COVID-19 patients. *Sci Rep.* (2021) 11:14042. 10.1038/s41598-021-93546-5 34234251 PMC8263555

[29] Farshidfar F Koleini N Ardehali H. Cardiovascular complications of COVID-19. *JCI Insight.* (2021) 6:e148980. 10.1172/jci.insight.148980 34061779 PMC8410051

[30] Johansson M Stahlberg M Runold M Nygren-Bonnier M Nilsson J Olshansky B Long-haul post-COVID-19 symptoms presenting as a variant of postural orthostatic tachycardia syndrome: the swedish experience. *JACC Case Rep.* (2021) 3:573–80. 10.1016/j.jaccas.2021.01.009 33723532 PMC7946344

[31] Chadda KR Ajijola OA Vaseghi M Shivkumar K Huang CL Jeevaratnam K. Ageing, the autonomic nervous system and arrhythmia: From brain to heart. *Ageing Res Rev.* (2018) 48:40–50. 10.1016/j.arr.2018.09.005 30300712

[32] Fu Q Vangundy TB Galbreath MM Shibata S Jain M Hastings JL Cardiac origins of the postural orthostatic tachycardia syndrome. *J Am Coll Cardiol.* (2010) 55:2858–68. 10.1016/j.jacc.2010.02.043 20579544 PMC2914315

[33] Bryarly M Phillips LT Fu Q Vernino S Levine BD. Postural orthostatic tachycardia syndrome: JACC focus seminar. *J Am Coll Cardiol.* (2019) 73:1207–28. 10.1016/j.jacc.2018.11.059 30871704

[34] Raj SR. The postural tachycardia syndrome (POTS): pathophysiology, diagnosis & management. *Indian Pacing Electrophysiol J.* (2006) 6:84–99. 16943900 PMC1501099

[35] Manger WM Eisenhofer G. Pheochromocytoma: diagnosis and management update. *Curr Hypertens Rep.* (2004) 6:477–84. 10.1007/s11906-004-0044-2 15527694

[36] Schondorf R Low PA. Idiopathic postural orthostatic tachycardia syndrome: an attenuated form of acute pandysautonomia? *Neurology.* (1993) 43:132–7. 10.1212/wnl.43.1_part_1.132 8423877

[37] Baig AM Khaleeq A Ali U Syeda H. Evidence of the COVID-19 virus targeting the CNS: tissue distribution, host-virus interaction, and proposed neurotropic mechanisms. *ACS Chem Neurosci.* (2020) 11:995–8. 10.1021/acschemneuro.0c00122 32167747

[38] Rysz S Al-Saadi J Sjöström A Farm M Campoccia Jalde F Plattén M COVID-19 pathophysiology may be driven by an imbalance in the renin-angiotensin-aldosterone system. *Nat Commun.* (2021) 12:2417. 10.1038/s41467-021-22713-z 33893295 PMC8065208

[39] Szabo GT Kiss A Csanadi Z Czuriga D. Hypothetical dysfunction of the epithelial sodium channel may justify neurohumoral blockade in coronavirus disease 2019. *ESC Heart Fail.* (2021) 8:171–4. 10.1002/ehf2.13078 33205539 PMC7753344

[40] Fu Q Shook RP Shibata S Hastings JL Okazaki K Conner CL *Vasomotor Sympathetic and Hemodynamic Responses During Upright Tilt in the Postural Orthostatic Tachycardia Syndrome.* Hoboken, NJ: Wiley Online Library (2007).

[41] Hoad A Spickett G Elliott J Newton J. Postural orthostatic tachycardia syndrome is an under-recognized condition in chronic fatigue syndrome. *QJM.* (2008) 101:961–5. 10.1093/qjmed/hcn123 18805903

[42] Miwa K Fujita M. Small heart syndrome in patients with chronic fatigue syndrome. *Clin Cardiol.* (2008) 31:328–33. 10.1002/clc.20227 18636530 PMC6653127

[43] Guedj E Million M Dudouet P Tissot-Dupont H Bregeon F Cammilleri S F-FDG brain PET hypometabolism in post-SARS-CoV-2 infection: substrate for persistent/delayed disorders? *Eur J Nucl Med Mol Imaging.* (2021) 48:592–5. 10.1007/s00259-020-04973-x 32728799 PMC7391029

[44] Swai J Hu Z Zhao X Rugambwa T Ming G. Heart rate and heart rate variability comparison between postural orthostatic tachycardia syndrome versus healthy participants; a systematic review and meta-analysis. *BMC Cardiovasc Disord.* (2019) 19:320. 10.1186/s12872-019-01298-y 31888497 PMC6936126

[45] Williams DP Koenig J Carnevali L Sgoifo A Jarczok MN Sternberg EM Heart rate variability and inflammation: A meta-analysis of human studies. *Brain Behav Immun.* (2019) 80:219–26. 10.1016/j.bbi.2019.03.009 30872091

[46] Blitshteyn S. Autoimmune markers and autoimmune disorders in patients with postural tachycardia syndrome (POTS). *Lupus.* (2015) 24:1364–9. 10.1177/0961203315587566 26038344

[47] Wu J Tang Y. Revisiting the immune balance theory: a neurological insight into the epidemic of COVID-19 and its alike. *Front Neurol.* (2020) 11:566680. 10.3389/fneur.2020.566680 33178109 PMC7593407

[48] Li H Yu X Liles C Khan M Vanderlinde-Wood M Galloway A Autoimmune basis for postural tachycardia syndrome. *J Am Heart Assoc.* (2014) 3:e000755. 10.1161/JAHA.113.000755 24572257 PMC3959717

[49] Sandroni P Low PA. Other autonomic neuropathies associated with ganglionic antibody. *Auton Neurosci.* (2009) 146 (1–2):13–7. 10.1016/j.autneu.2008.10.022 19058765 PMC2671239

[50] Kavi L Gammage MD Grubb BP Karabin BL. Postural tachycardia syndrome: multiple symptoms, but easily missed. *Br J Gen Pract.* (2012) 62(599):286–7. 10.3399/bjgp12X648963 22687203 PMC3361090

[51] O’Sullivan JS Lyne A Vaughan CJ. COVID-19-induced postural orthostatic tachycardia syndrome treated with ivabradine. *BMJ Case Rep.* (2021) 14:e243585. 10.1136/bcr-2021-243585 34127505 PMC8204164

[52] Sheldon RS Grubb BP II Olshansky B Shen WK Calkins H Brignole M 2015 heart rhythm society expert consensus statement on the diagnosis and treatment of postural tachycardia syndrome, inappropriate sinus tachycardia, and vasovagal syncope. *Heart Rhythm.* (2015) 12:e41–63. 10.1016/j.hrthm.2015.03.029 25980576 PMC5267948

[53] Jeevaratnam K. Chloroquine and hydroxychloroquine for COVID-19: implications for cardiac safety. *Eur Heart J Cardiovasc Pharmacother.* (2020) 6:256–7. 10.1093/ehjcvp/pvaa041 32347923 PMC7197554

[54] Thieben MJ Sandroni P Sletten DM Benrud-Larson LM Fealey RD Vernino S Postural orthostatic tachycardia syndrome: the Mayo clinic experience. *Mayo Clin Proc.* (2007) 82:308–13. 10.4065/82.3.308 17352367

